# Profiles of parent–teacher discrepancy on autistic children’s adaptive functioning

**DOI:** 10.1177/13623613251407310

**Published:** 2026-01-07

**Authors:** Rachel Lees Thorne, Nicky Wright, Andres De Los Reyes, Isabel M Smith, Anat Zaidman-Zait, Lonnie Zwaigenbaum, Tracy Vaillancourt, Peter Szatmari, Teresa A Bennett, Eric Duku, Annie E Richard, Connor Kerns, Rachael Bedford, The Pathways in ASD Study Team

**Affiliations:** 1 University of Bath, UK; 2 Manchester Metropolitan University, UK; 3 University of Maryland, USA; 4 Dalhousie University, Canada; 5 IWK Health Centre, Halifax, Canada; 6 Tel Aviv University, Israel; 7 University of Alberta, Canada; 8 University of Ottawa, Canada; 9 The Hospital for Sick Children, Canada; 10 University of Toronto, Canada; 11 McMaster University, Canada; 12 University of British Columbia, Canada; 13 Queen Mary University of London, UK

**Keywords:** adaptive functioning, executive function, latent profile analysis, multi-informant

## Abstract

Clinical guidelines recommend collecting reports from multiple informants when identifying and diagnosing challenges in children. The current study examined parent–teacher discrepancies in rating of autistic children’s adaptive functioning and how these related to children’s executive functions. Participants (*n* = 194) were a subsample of autistic children (mean age = 9.2 years; 86% male) from the *Pathways in ASD* cohort. We used latent profile analysis to characterise profiles based on both parent and teacher reports of adaptive functioning levels. We tested links between these profiles and indices of children’s executive function and other clinical correlates. Four profiles were characterised: a *lower adaptive functioning-parent higher* profile, in which parents reported relatively higher scores than teachers (*n* = 45), an *intermediate adaptive functioning* profile (*n* = 70) and a *higher adaptive functioning* profile (*n* = 39; both characterised by similar ratings between informants) and finally, a *higher adaptive functioning-teacher higher* profile, in which teachers reported relatively higher scores than parents (*n* = 40). The *higher adaptive functioning-teacher higher* profile showed fewer teacher-rated executive function challenges and higher IQ compared to the other profiles. Characterising profiles facilitates interpretation of informant discrepancies and identification of clinical correlates to inform clinical decision-making.

**Lay abstract:**

Clinicians are advised to collect reports from multiple informants (e.g., parents and teachers), when making assessments about the wellbeing of autistic children. Parents and teachers observe children in different environments (home vs. school); therefore, collecting both reports can give a fuller account of a child’s strengths and challenges. In this investigation, we looked at parent and teacher reports of autistic children’s adaptive functioning, an important body of skills necessary for children to navigate daily life including practical, communication and conceptual skills. Currently, we know little about child characteristics associated with informant discrepancies, which means that it is a challenge to identify which children are most likely to display behaviour differently across contexts. We grouped *n* = 194 children based on the level of adaptive functioning reported by both their parent and teachers, and we compared the groups on key characteristics. We identified four groups: a lower adaptive functioning group with higher parent scores (*n* = 45), an intermediate group with similar scores from both informants (*n* = 70), a higher adaptive functioning group with similar scores from both informants (*n* = 39) and a higher adaptive functioning group with higher teacher scores (*n* = 40). Our findings indicate that many children display adaptive functioning skills differently across contexts, across levels of adaptive functioning skills. We found that children across groups differed on IQ, autistic traits and teacher-rated executive functioning. These findings can help clinicians identify and evaluate autistic children that might be likely to demonstrate different adaptive functioning skills in different environments, which could help with assessment and treatment planning.

## Introduction

Autism is a neurodevelopmental condition characterised by differences in social communication as well as restricted or repetitive interests and behaviour that occurs in approximately 1%–2% of children (Baio et al., 2018). The clinical profile of autistic children is heterogeneous, with variability in the presentation of core autistic traits, mental health comorbidities and adaptive functioning. Adaptive functioning refers to the body of skills necessary to navigate daily life by meeting the demands of the environment through practical, communication and conceptual skills. Autistic children tend to have greater challenges with their adaptive functioning, regardless of cognitive function or IQ level, than typically developing children (Pugliese et al., 2016), indicating this as a key facet of autism presentation. However, autistic children vary considerably in levels of adaptive functioning, and this variation can influence treatment planning (Farmer et al., 2018; Taylor & Henninger, 2015). Understanding the factors which contribute to variation in adaptive function, and its presentation across different settings, is important for providing targeted support for autistic children.

Clinical guidelines indicate that both parent and teacher reports should be used in the assessment of adaptive functioning (De Los Reyes et al., 2022). These reporters interact with children in different contexts (i.e., home vs. school), and these contexts may vary in the demands they place on children (Kraemer et al., 2003). Thus, acquiring reports of adaptive functioning skills across settings provides a well-rounded view of how a child is coping and where they require support. In line with this assessment approach, studies typically reveal low-to-moderate correlations between parent and teacher reports of autistic children’s adaptive functioning (Jordan et al., 2019; McDonald et al., 2016; Moore et al., 2023; Stevens et al., 2023). Studies vary as to whether parents or teachers rate children as having higher adaptive functioning (cf. Dickson et al., 2018; Jordan et al., 2019; McDonald et al., 2016; Moore et al., 2023; Stevens et al., 2023). Furthermore, parent–teacher discrepancies may vary based on the overall level of the adaptive functioning of the child, with greater discrepancy at higher levels of adaptive function (McDonald et al., 2016; Moore et al., 2023; Stevens et al., 2023), although one investigation found discrepancy was not related to level of adaptive functioning (Jordan et al., 2019). The variability observed across studies could be due to clinically meaningful subgroups existing within studies that could reveal different patterns of parent–teacher agreement/discrepancy across the spectrum of adaptive abilities.

Identifying factors, including child characteristics, that are associated with reporter discrepancy could help when assessing and diagnosing autism and co-occurring conditions in children. For example, parent–teacher agreement on child autistic traits has been associated with medication status, clinician-rated autistic traits and special education support (Kang et al., 2023; Lerner et al., 2017). However, studies have found no evidence for a moderating role of autistic traits, age, IQ or sex on parent–teacher agreement on adaptive functioning (Dickson et al., 2018; Jordan et al., 2019; McDonald et al., 2016; Moore et al., 2023; Stevens et al., 2023). Studies that have revealed null effects typically employed difference scores to measure informant agreement and its association with other child characteristics, an approach that comes with interpretive challenges (Laird & Weems, 2011). Difference scores do not allow their users to test whether the ‘space’ between informants’ reports yields incrementally valuable data that cannot be obtained from one or both informants, nor do they maintain information on the level of the construct measures (De Los Reyes et al., 2023; Laird & De Los Reyes, 2013). In the current study, we address this by taking a person-centred approach to characterise subgroups of children based on both parent and teacher reports of adaptive function. We also test links between these profiles and clinical characteristics. Thus, we intend to characterise profiles of agreement/discrepancy between parent and teacher reports, as well as test the validity and clinical value of these profiles.

One key factor that may contribute to not only a child’s adaptive functioning but also their adaptability across settings, and thus informant agreement, is executive functions (EFs), a suite of cognitive abilities including working memory, planning, inhibition and cognitive flexibility (Kenny et al., 2019; Pugliese et al., 2016). EF in autistic children is commonly assessed using the Behavior Rating Inventory of Executive Function (BRIEF), a questionnaire measure of real-world EF and related behaviour. The BRIEF comprises two higher-order components: metacognition and behaviour regulation (Gioia et al., 2000). Autistic children may display challenges with these skills (Demetriou et al., 2018). Furthermore, metacognition and behaviour regulation have both been associated with adaptive functioning in autistic children (Gardiner & Iarocci, 2018; Pugliese et al., 2015, 2016; Wallace et al., 2016).

EF skills are relevant to the study of informant discrepancies on the reporting of autistic children because individuals with stronger EF skills might be better able to modify their behaviour in different contexts (Cook et al., 2021; Livingston et al., 2019). Therefore, variability in levels of informant discrepancy may be linked to variations in EF skills. Thus, we expect that for a subgroup of children with greater EF skills, we could observe greater informant discrepancies. As the school environment can be particularly challenging for autistic children (Ashburner et al., 2010), we expect that children who are rated as having stronger adaptive functioning by teachers compared to parents will also have stronger EF.

The current study uses data from the *Pathways in ASD* study, which has followed an inception cohort of autistic children. Adaptive functioning and EF were rated by both parents and teachers when children were approximately 8.5 years old and again at approximately age 10.5 years. The aims of the current study are (1) to characterise profiles of parent–teacher discrepancies in adaptive functioning in a cohort of autistic children; (2) to compare profiles on background and clinical characteristics, including mental health, autistic traits and IQ and (3) to test whether profiles vary in EF. We predicted that we would identify profiles that differed based on levels of both parent- and teacher-rated adaptive functioning, as well as whether parents and teachers gave similar ratings. Furthermore, we hypothesised that EFs would be highest in profiles of children who were rated as having stronger adaptive functioning by both informants compared to those with lower adaptive functioning and in those profiles rated as relatively stronger by teachers compared to parents. As this analytic approach is data driven, we were unable to make hypotheses based on specific profiles before conducting the analysis.

## Method

### Study population

Inclusion criteria for the *Pathways in ASD* study were (1) aged between 2 years and 4 years and 11 months at the time of diagnosis, (2) met criteria for ASD based on the Autism Diagnostic Observation Schedule (ADOS) and Socialisation and one other domain of the Autism Diagnostic Interview – Revised (ADI-R; Risi et al., 2006) and (3) met the *Diagnostic and Statistical Manual of Mental Disorders* (4th ed.; DSM-IV) criteria for ASD according to qualified clinicians. Exclusion criteria were known genetic or chromosomal abnormalities, neuromotor disorders, severe hearing and/or visual impairments. Four hundred and twenty-one children met criteria to take part upon entry into the cohort, which occurred within 4 months of an autism diagnosis. The *Pathways in ASD* study involved two phases across early and middle childhood. In Phase 1 (child age 2–6 years), demographic data were collected as well as clinical assessment of autistic traits and other performance measures. Phase 2 (age 7–11 years) corresponded with the children becoming of school age, at which point teacher assessments of child wellbeing were collected alongside parent report. Parent and teacher reports of adaptive functioning and EF were completed when children were approximately 8.5 years and again at approximately 10.5 years. Children with concurrent parent and teacher reports of their adaptive functioning on at least one of these occasions were included in the current analysis (see section ‘Analytic plan’ for further detail). This resulted in a sample size of *n* = 194. The *Pathways in ASD* study recruited children based on consecutive referrals within specified geographic regions across five Canadian provinces. The study was approved by the local research ethics boards at all recruitment sites, and families gave written informed consent for their children to participate.

### Community involvement

In 2005, parents, advocates, researchers and practitioners met to establish the aims of the *Pathways in ASD* study. Community members continue to engage in aspects of the study, including providing feedback on study protocols and research aims and informing the design of Phase 2. Community members were not directly involved in shaping the research questions, analytic approach and interpretation of results for the current study.

### Measures

#### Adaptive function

The Vineland Adaptive Behavior Scales II (VABS-II; Sparrow et al., 2005) were completed by both parent and teacher to assess adaptive behaviour evidenced in the home and school context, respectively. Parents completed the assessment in a semi-structured interview format, and teachers completed a questionnaire. The items across assessments reflect appropriate adaptive behaviours across the home versus school environment. Many items overlap exactly between the informant reports, whereas some are more environment-specific (e.g., household chores vs. following rules in the classroom). Items are rated on a 3-point scale: 0 = *behaviour never performed*, 1 = *behaviour sometimes performed*, 3 = *behaviour usually or habitually performed*. Three validated subscales include Communication (Receptive, Expressive and Written), Daily Living Skills (personal, domestic/academic and community) and Socialisation (interpersonal relationships, play and leisure time and coping skills). We used standard scores; higher scores indicate better adaptive functional ability. The VABS-II has been demonstrated to have good concurrent validity and reliability (Sparrow et al., 2005) and has been commonly used in studies of autistic populations.

#### EF

Parents and teachers completed the BRIEF 5–18 (Gioia et al., 2000). Informants complete the assessment based on their observation of behavioural manifestations of EF in the relevant context, for example, home and school. Items are rated on a 3-point scale: 1 = *Never*, 2 = *Sometimes* and 3 = *Often*. This 86-item assessment generates eight clinical scales that make up two validated higher-order subscales: Metacognition (initiate, working memory, planning, task monitor and organisation subscales) and Behavioural Regulation (inhibit, shift and emotional control subscales). Satisfactory internal consistency and test–retest reliability has been evidenced for the BRIEF (Gioia et al., 2000). The BRIEF is commonly used as a measure of daily life EF behaviour in autistic children (Demetriou et al., 2018). Here, we used raw scores, with higher scores indicating greater EF difficulties.

#### Clinical characteristics

*Mental Health*. Parents completed the Child Behavior Checklist (CBCL/6–18), and teachers completed the Teacher Report Form. These complementary measures give rise to two subscales, representing problems with internalising behaviours (anxious/depressed, withdrawn/depressed and somatic complaints) and externalising behaviours (rule-breaking and aggressive behaviour). The CBCL has demonstrated validity in the measurement of emotional and behavioural problems in school-aged autistic children (Pandolfi et al., 2012). We used standard scores, with higher scores indicating greater difficulties.

*Autistic traits*. The ADOS (Lord et al., 2000), a semi-structured assessment of autistic traits, was conducted by a research-reliable examiner with participants at approximately age 6.5 years. We used ADOS calibrated severity scores (Gotham et al., 2009) as a measure of autistic trait levels that is independent of the informants who provided reports for creating profiles of agreement/discrepancy (i.e., parents and teachers). In this respect, use of an independent measure like the ADOS is in keeping with recommendations for interpreting profiles of agreement/discrepancy (i.e., to rule out shared method biases as explaining all observed effects) (De Los Reyes et al., 2023).

Background characteristics. Other relevant characteristics include child sex assigned at birth, recruitment site (Halifax, Montreal, Hamilton, Edmonton, Vancouver), child age at assessment (in months) and socioeconomic status, measured via household income. This was captured on a scale of 1 (<$5,000) to 11 (>*$80,000*). We dichotomised this variable at ⩽$40,000 and >$40,000 to identify the extreme 15% of the distribution towards the lower end of the income range, as has been done in previous studies (Baribeau et al., 2020; Browne et al., 2016; Zaidman-Zait et al., 2018). Finally, a subset of children completed the Wechsler Intelligence Scale for Children (WISC-IV; Wechsler, 1949) at approximately 8.5 years; here, we use the full-scale composite score.

#### Analytic plan

Parent and teacher ratings on the three VABS-II subscales were used as indicators in a latent profile analysis (LPA), using Mplus Version 8 (Muthén & Muthén, 2009). LPA is a person-centred statistical approach in which profiles are determined based on groupings of similar responses on the indicator variables. The model is run repeatedly, increasing the number of latent profiles each time. The optimal number of latent profiles was determined by comparing model fit across five models (see Supplementary Table 1). Fit statistics included the sample-adjusted Bayesian information criterion (aBIC), the Lo–Mendel–Rubin likelihood ratio test (LMR-LRT) and the Bootstrapped likelihood ratio test (BLRT; Nylund et al., 2007). Furthermore, we considered the proportion of individuals in each class, entropy (where higher entropy values indicate better classification), parsimony and the interpretability of the classes. As the Pathways cohort recruited children across multiple sites in Canada, site was included as a covariate in the LPA model. Participants were included in the LPA analysis if they had concurrent parent and teacher reports of their adaptive functioning at either age 8.5 or age 10.5 years. We prioritised data from the age 8.5 assessment, for which more data were available (*n* = 148). For children without data at this time point, we used data from the assessment at age 10.5 years (*n* = 46). We included a binary covariate in the LPA model to indicate at which time point the child was assessed. In addition, BRIEF and CBCL data that was used to compare the profiles was selected to match the time point at which VABS was assessed, to maximise data availability and maintain consistency across assessments. Children in the analytic sample (*n* = 194) did not differ significantly from children excluded for missing VABS-II data (*n* = 226) on sex (X^2^ = .28, *p* = .597), recruitment site (X^2^ = 2.64, *p* = .619) or socioeconomic status (X^2^ = .27, *p* = .602). There was a significant difference between the analytic sample and the excluded sample on ADOS calibrated total severity scores (mean difference = −.66, *p* = .007), indicating lower levels of autistic characteristics in the excluded sample. Age of diagnosis was also significantly younger in the excluded compared to the analytic sample (mean diff = −2.18 months, *p* = .010).

Latent profiles were compared on background and clinical characteristics using analyses of variance (ANOVAs) and chi-square tests, as appropriate. We used a stepwise approach to determine whether to conduct follow-up contrasts from these summary statistics (using a Holm–Bonferroni correction). This analysis plan was chosen based on the exploratory nature of these comparisons. The association between EF and patterns of parent–teacher adaptive behaviour reports was estimated using multinomial logistic regression, with latent profile as the outcome. This approach was selected primarily to compare the *higher AF-teacher higher* profile with the *higher AF* profile, which had similar parent ratings but differing teacher reports. It also allowed us to include relevant covariates, in line with our hypothesis-testing framework. Each participant was assigned to their ‘most likely’ profile based on the model-estimated posterior probabilities, an appropriate approach when model entropy indicates good separation between classes, as was the case here (Sinha et al., 2021). Teacher and parent reports on the BRIEF (metacognitive and behavioural regulation subscales) were entered into separate models as predictors. Covariates in regression models included child sex, age and socioeconomic status. Due to missing data on socioeconomic status (ranging from 23% to 31% across latent profiles) and the availability of auxiliary variables that were well correlated with socioeconomic status for imputation of this variable, we imputed missing values using chained equations in STATA (see Supplementary Materials for further details).

## Results

**Figure 1.**
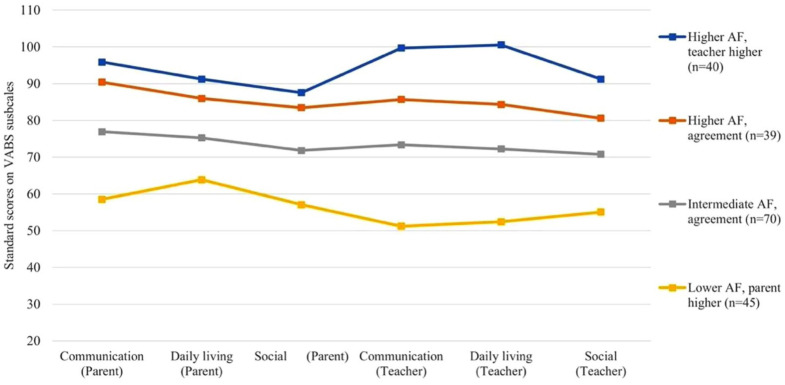
Latent profiles of parent and teacher means on subscales of the Vineland Adaptive Behavior Scales II.

Table 1 shows the correlations between parent and teacher reports on VABS-II subscales, which were all positively and strongly (>.60) correlated. A four-profile solution was chosen as the best model, based on model fit statistics and interpretability (see Figure 1 and Supplementary Table 1). Covariates of assessment timepoint and site were not significant in the LPA model (see Supplementary Table 2). This solution identified classes across the range of adaptive function levels, as well as classes in which parents and teachers either reported similar or dissimilar mean scores. Therefore, despite the LMR-LRT test not indicating a significantly greater fit in the four-class compared to the three-class solution, we opted for the four-class model for interpretability. Entropy levels were high across all models. We identified a *lower AF-parent higher* profile (*n* = 45, 23%), for whom parents reported higher skills than teachers (mean diff = 6.67, *t* = 5.86, *p* < .001). A profile indicated *intermediate AF* (*n* = 70, 36%), with a smaller but significantly different discrepancy with parents scoring higher than teachers (mean diff = 2.40, *t* = 2.13, *p* = .036). Finally, we identified two profiles of *higher adaptive functioning* with discrepant patterns with regard to parent/teacher discrepancy. In one *higher AF* profile (*n* = 39, 20%), differences between parent and teacher means were small but significant, with parents scoring higher (mean diff = 3.38, *t* = 2.22, *p* = .032). In the final *higher AF* profile (*n* = 40, 21%), teachers reported higher scores than parents (mean diff = 5.68, *t* = 3.06, *p* = .004). After applying a Holm–Bonferroni correction, only the *lower AF-parent higher-*profile and *higher AF-teacher higher-*informant differences remain statistically significant; therefore, these profiles are referred to as discrepancy classes.

**Table 1.** Correlations between parent and teacher reports on VABS-II, BRIEF and CBCL.

Assessment scale	Pearson’s *r*	*p*
VABS communication	.80	< .001
VABS daily living	.64	< .001
VABS socialisation	.69	< .001
BRIEF metacognition	.34	< .001
BRIEF behaviour regulation	.33	< .001
CBCL externalising	.31	< .001
CBCL internalising	.18	.022

VABS = The Vineland Adaptive Behavior Scales II, BRIEF = Behavior Rating Inventory of Executive Function 5–18, CBCL = Child Behavior Checklist (CBCL/6–18).

### Background and clinical characteristics across profile

The profile of children with *lower AF-parent higher* had a mean cognitive age of 28 months (estimated using the Merrill–Palmer–Revised Scales of Development: Developmental Index). The minimum age for assessment of EF using the BRIEF is 5 years (Gioia et al., 2000); therefore, we do not include statistical comparisons between this profile and the others on EF. Furthermore, due to a large amount of missing data on the WISC in this *lower AF-parent higher* profile, we do not include them in profile comparisons of IQ.

Sample characteristics across profiles are described in Table 2. Profiles differed based on IQ, driven by significantly lower IQ in the *intermediate* compared to both *higher AF* profiles, as well as significantly lower IQ in the *higher AF* group compared to the *higher AF-teacher higher* profile. ADOS scores also differed across profiles, with significantly lower scores in the *high AF-teacher higher* profile compared to the *lower AF-parent higher* and *intermediate AF* profiles. The *higher AF* profile also had significantly lower ADOS scores than the *intermediate AF* profile. Teacher-rated externalising behaviour also differed, with significantly lower scores in the *higher AF* profiles compared to the *intermediate AF* and *low AF* profiles and significantly lower scores in the *intermediate AF* profile compared to *low AF*. Finally, socioeconomic status differed across profiles, with significantly more families earning less than 40k CAD per annum in the *lower AF-parent higher* profile compared to all other profiles.

**Table 2.** Sample characteristics.

Variable	Lower AF-parent higher (*n* = 45)	Intermediate AF (*n* = 70)	Higher AF (*n* = 39)	Higher AF-teacher higher (*n* = 40)	Profile comparisons
Sex, male:female	35:10 *n* = 45	62:8 *n* = 70	35:4 *n* = 39	34:6 *n* = 40	X^2^(3) = 3.28, *p* = .350
Age in months, *M* (*SD*)	108.27 (8.67) *n* = 45	110.34 (10.61) *n* = 70	111.22 (11.82) *n* = 39	112.03 (11.66) *n* = 40	*F*(3, 190) = 0.99, *p* = .400
Ethnic background, white:other ethnic background	22:17 *n* = 39	52:13 *n* = 65	30:8 *n* = 38	31:9 *n* = 40	X^2^(3) = 8.30, *p* = .041
Household income Above:below 40k p/a	18:13 *n* = 31	45:11 *n* = 56	25:4 *n* = 29	29:3 *n* = 32	X^2^(3) = 11.79, *p* = .008^a^
ADOS total calibrated severity score, *M* (*SD*)	7.51 (1.32) *n* = 43	7.87 (1.55) *n* = 70	6.92 (2.36) *n* = 39	6.24 (2.19) *n* = 38	*F*(3, 186) = 7.24, *p* < .001^a^
WISC (IV) full-scale composite score, *M* (*SD*)	52.00 (0) *n* = 2	72.29 (12.60) *n* = 48	86.65 (15.71) *n* = 37	95.64 (18.09) *n* = 39	*F*(2, 121) = 25.49, *p* < .001^a^ *Lower AF group excluded*
Parent CBCL externalising, *M* (*SD*)	56.67 (9.84) *N* = 30	51.64 (11.78) *N* = 59	50.55 (11.51) *N* = 33	48.17 (8.91) *N* = 35	*F*(3, 153) = 3.49, *p* = .017
Teacher TRF externalising, *M* (*SD*)	63.33 (6.63) *N* = 43	58.67 (7.94) *N* = 67	55.29 (7.84) *N* = 38	49.88 (6.38) *N* = 40	*F*(3, 184) = 25.14, *p* < .001^a^
Parent CBCL internalising, *M* (*SD*)	55.27 (9.70) *N* = 30	53.95 (9.20) *N* = 59	54.03 (12.19) *N* = 33	54.17 (11.84) *N* = 35	*F*(3, 153) = 0.11, *p* = .952
Teacher TRF internalising, *M* (*SD*)	59.23 (6.98) *N* = 43	59.99 (9.16) *N* = 67	58.53 (8.71) *N* = 38	56.02 (8.08) *N* = 40	*F*(3, 184) = 1.94, *p* = .124

ADOS = Autism Diagnostic Observation Schedule, SES = socioeconomic status, WISC = Wechsler Intelligence Scale for Children, CBCL = Child Behaviour Checklist, TRF = Teacher Report Form. Missing data: Missing values on socioeconomic status were imputed using chained equations up to the full analysis sample (*n* = 194). Data in Table 2 show the observed (not imputed values). See the supplement for further details on missing data and imputation.

a Group comparison *p*-value was significant after the Holm–Bonferroni correction, so follow-up contrasts were conducted.

### EF regression analyses

EF scores across the profiles are displayed in Table 3. The *higher AF-teacher higher* profile was chosen as the reference category in multinomial logistic regression models, as the comparison of this profile with the *higher AF* profile was of primary interest due to their similar parent ratings across all subscales despite diverging teacher reports.

**Table 3.** EF (BRIEF) scores across profiles.

BRIEF subscale	Lower AF^a^	Intermediate AF (*n* = 70) *M* (*SD*)	Higher AF (*n* = 39) *M* (*SD*)	Higher AF-teacher higher (*n* = 40) *M* (*SD*)
Parent metacognition	n/a	95.21 (16.54) *N* = 58	83.77 (20.24) *N* = 31	80.86 (16.96) *N* = 36
Teacher metacognition	n/a	95.75 (18.14) *N* = 68	84.95 (17.98) *N* = 39	68.70 (16.30) *N* = 40
Parent behaviour regulation	n/a	55.19 (10.65) *N* = 58	50.74 (11.77) *N* = 31	47.92 (10.76) *N* = 36
Teacher behaviour regulation	n/a	59.60 (14.24) *N* = 70	52.95 (11.02) *N* = 39	43.10 (9.52) *N* = 40

a BRIEF assessment in the *lower AF* profile was deemed inappropriate due to a low mean cognitive age of participants in this profile; therefore, this profile was excluded from comparisons of executive function.

Analyses indicated that both metacognition and behavioural regulation as measured via BRIEF teacher report were associated with profile membership (see Table 4 for statistical details of EF analyses). The *higher AF* profile had worse metacognitive scores and lower behavioural regulation than the *higher AF-teacher higher* profile. Participants in the *intermediate AF* profile also evidenced weaker metacognition and behavioural regulation scores than the *higher AF-teacher higher* profile.

**Table 4.** Model results for executive functioning across *intermediate* and *higher AF* profiles.

	Intermediate AF		Higher AF		Higher AF-teacher higher
BRIEF subscale	Effect (95% CIs)	*p*	Effect (95% CIs)	*p*	Reference profile
Parent metacognition	.05 (.02, .07)	*p* *=* .001	.01 (-.02, .040)	*p* *=* .499	-
Parent behavioural regulation	.06 (.02, .11)	*p* *=* .005	.03 (-.02, .07)	*p* *=* .281	-
Teacher metacognition	.09 (.06, .12)	*p* < .001	.06 (.03, .09)	*p* < .001	-
Teacher behavioural regulation	.12 (.08, .17)	*p* < .001	.08 (.03, .12)	*p* *<* .001	-

CIs = confidence interval.

*Note:* BRIEF assessment in the *lower AF* profile was deemed inappropriate due to a low mean cognitive age of participants in this profile; therefore, this profile was excluded from comparisons of executive function.

Using parent-reported BRIEF scores, no statistically significant difference was evidenced between the *higher AF* profile and the *higher AF-teacher higher* profile on metacognition or behavioural regulation. Participants in the *intermediate AF* profile had weaker parent-reported metacognition and behavioural regulation scores than the *higher AF-teacher higher profile*.

## Discussion

Comprehensive measurement of children’s adaptive functioning relies on collecting data from multiple informants. Most commonly, adaptive functioning is reported on by parents and teachers, and because these informants observe behaviour in different contexts, studies frequently reveal discrepancy in reports. The present study used a person-centred statistical approach to examine profiles of agreement/discrepancy in parent and teacher reports of adaptive functioning in autistic children and clinical correlates of these profiles. Four profiles were identified that differed based on the overall level of the child’s adaptive functioning, with some profiles demonstrating differences in parent and teacher reports. The profiles also differed across several clinical characteristics, including teacher (but not parent)-rated metacognition and behavioural regulation, externalising behaviour symptoms, as well as children’s IQ and ADOS scores. These findings suggest that domains such as child EF and IQ may influence the presentation and/or observation of adaptive functioning across environments.

Our analysis approach extends previous research on adaptive functioning by considering the presence of clinically meaningful profiles within our overall sample of autistic children. Previous findings have been inconsistent, with some studies finding that parents rate autistic children’s adaptive functioning as higher than do teachers, and others finding the opposite. Yet, recent work reveals substantial variability *within studies* using parent and teacher reports to assess autistic children (Kang et al., 2023; Lerner et al., 2017). In line with prior work, we characterised discrepancy across levels of adaptive functioning abilities and found evidence for different directions of discrepancy within the sample. Whereas teachers reported greater adaptive strengths in some children (*higher AF-teacher higher*, *n* = 39), parents indicated greater adaptive strengths in others (*lower AF-parent higher*, *n* = 45). Two profiles were characterised by similar mean scores across parents and teachers across all subscales: *intermediate AF* and *higher AF* groups (*n* = 109).

Overall, the identified profiles support the notion that reports from both teachers and parents yield a broader picture of a child’s adaptive functioning than relying on one of these informants alone. For example, parent-reported adaptive function levels were similar across the *higher AF-teacher higher* and *higher AF profiles*; however, teacher reports suggested that these groups displayed different levels of adaptive function in the classroom environment. Moreover, for children in the lower adaptive functioning range, parent report may not indicate teacher report, particularly for behaviours such as communication and daily living skills. This suggests that even at lower levels of adaptive functioning, children may evidence variable presentations across contexts, and having the perspective of a teacher could give a fuller account for these children and may uncover challenges as well as strengths that are not observed in the home environment.

Notably, all subgroups evidenced some level of discrepancy in mean scores between parent and teacher across the VABS subscales, with the degree and direction of discrepancy differing across classes. In classes with evidence of greater informant discrepancy (*higher AF-teacher higher, lower AF-parent higher)*, parents and teachers showed the greatest discrepancies in their reports of children’s daily living skills, although discrepancies were not exclusive to this subscale. The *lower AF-parent higher* group evidenced parents reporting significantly higher skills overall and on all subscales related to communication and daily living. The *higher AF-teacher higher* group showed significantly better daily living skills according to teacher reports compared to parent reports, with trend-level differences observed in the communication (*p* = .057) and social subscales (*p* = .056). Daily living skills encompass behaviour such as eating and drinking, dressing, household chores and counting, as well as understanding of abstract concepts such as time and mathematics. These skills are essential for the ability to live independently; therefore, this discrepancy is important for the clinical assessment of adaptive function. Of note, parents reported on ‘domestic’ daily living skills (e.g., kitchen chores, housekeeping), and teachers reported on ‘academic’ daily living skills (e.g., mathematics, knowledge of money, understanding of time). Therefore, discrepancy in parent–teacher reports of daily living skills could stem from the inherent differences in these concepts as they are assessed. Regardless, it is important that the assessment of adaptive functioning is tailored to the environment in which the informant typically observes the child and has expertise, and picking up on these relative strengths or weaknesses in the school environment provides important insight into where children display appropriate adaptive abilities.

Our identified agreement profiles differed across several demographic and clinical characteristics including EF, externalising behaviour, IQ, clinician-observed autistic traits and socioeconomic status. We found that the *higher AF-teacher higher* group had a higher mean IQ than the *higher AF* group. Teachers also rated these children as having better EF. This aligns with previous research demonstrating a relationship between adaptive function and IQ (Kenworthy et al., 2010) and may indicate that in the school environment in particular, greater IQ is positively associated with adaptive function. The correlates identified here could be relevant to the presentation of adaptive functioning across home and school environments in different ways. First, children with higher IQ and EF abilities may thrive in the school environment (Miller et al., 2017; Pellicano et al., 2017) and subsequently display fewer challenges than they do at home, leading to teachers observing fewer of these children’s adaptive function difficulties. Alternatively, teachers may view children with higher IQ as requiring less support, which could lead to them underestimating other challenges (such as adaptive function). These clinically relevant group differences support the use of this method in studies of informant agreement, aligning with other research using the same approach to characterise parent–teacher agreement on autistic traits (Kang et al., 2023; Lerner et al., 2017). Finally, the *lower AF* profile had significantly lower family income than all other profiles, as well as the highest externalising challenges. This could reflect a relationship between lower income and reduced access to services (McIntyre & Zemantic, 2017; Smith et al., 2020), highlighting how socioeconomic barriers could limit a child’s developmental potential, and thus emphasising the importance of expanding access to intervention and support in underserved communities. In addition, lower family income has been associated with lower accuracy of parent-completed developmental assessments, which could contribute to the discrepancy observed in this group (e.g., Herbers et al., 2017). Greater teacher-rated externalising challenges in the *lower AF* group may reflect their struggle to meet the demands of a school environment, which could lead to increased frustration displayed in this more complex and challenging setting.

Previous investigations have not identified reliable correlates of informant agreement on adaptive function. It could be that because these investigations have not typically considered the level of a child’s adaptive function when assessing agreement, which may have obscured the ability to detect predictors of agreement. For example, in children with generally higher adaptive functioning, greater IQ may lead to a better ability to modify behaviour across contexts (similar to our hypothesised role for EF), thus leading to more informant discrepancy. However, for children with lower adaptive functioning, greater IQ may lead to a child being relatively better at communicating their needs to parents and teachers, which could result in less discrepancy. Therefore, the correlates identified here are novel and warrant further research to fully characterise children who are more likely to display adaptive functioning differently across school and home.

We hypothesised that groups would differ on their EF based on previous research indicating EF is associated with adaptive functioning and might be associated with the ability to modify behaviour across contexts, which could lead to informants in different environments observing different levels of behaviour (Cook et al., 2021; Gardiner & Iarocci, 2018). Teachers, but not parents, rated metacognition and behaviour regulation as stronger in the higher adaptive function profile for whom teachers rated adaptive function as stronger than did parents, compared to the profile with comparable parent–teacher reports of higher adaptive function. This provides evidence in support of our hypothesis of a role of EF in contextual variation in symptoms. However, this may be due to a ‘halo’ effect (i.e., common source bias), whereby one informant is more likely to report similarly across several behaviours. Of note, parent-rated EF aligned in the same direction as teachers but did not reach statistical significance, and as mentioned previously, we did also observe differences among the profiles on independent assessments of key clinical correlates (i.e., ADOS, IQ), indicating that the profiles represent more than common informant bias. The current findings indicate that EF could be useful to consider in the evaluation of children with discrepant reports from multiple informants and may help to identify environments in which autistic children evidence particular strengths in their adaptive abilities.

Also of note, parent and teacher reports of adaptive functioning in the current study were strongly correlated (*r*s: 0.64–0.80) compared to their reports for EF (*rs*: .33–.34), externalising (*r* = .31) or internalising (*r* = .18). These findings could be attributed to the more observable nature of adaptive functioning or a larger environmental influence on the presentation of other behaviours, particularly internalising. Our adaptive functioning correlations are also higher than previously observed, although one study did find a high correlation between parent and teacher reports, particularly for the VABS-II Communication subscale (Dickson et al., 2018). Sample characteristics could be partially driving differences; most previous studies have been conducted in children enrolled in psychosocial interventions that employ an inclusion criterion based on IQ, typically >70 (Jordan et al., 2019; McDonald et al., 2016; Moore et al., 2023). It could be that the general level of cognitive functioning of the sample is related to parent–teacher discrepancy, given that previous research indicates greater informant disagreement at higher levels of adaptive functioning. Furthermore, while correlations indicate the strength of linear association between informants, this does not give insight into whether informants agree on the actual trait scores, underscoring the benefits of the latent profile approach.

Our findings should be considered in light of several limitations. Given the relatively modest sample size, we may have been underpowered to detect additional profiles with different patterns of symptoms. However, based on findings from their simulation study, Tein et al. (2013) suggest that factors such as the number of indicators, class proportions and model entropy play a more critical role than sample size alone in accurately identifying the correct number of classes. In the current study, we included six continuous indicators, observed a minimum class proportion of 20% and evidenced high model entropy (0.90), all of which support the robustness of our class solution. Nonetheless, the modest sample size warrants cautious interpretation and replication in larger samples. In addition, parents completed the VABS as an interview with a trained clinician, whereas teachers completed the VABS in questionnaire format. This is in line with previous published literature on VABS informant discrepancies (e.g., Moore et al., 2023); however, this does present some additional challenges in comparing these scores directly. In addition, this analysis is based on cross-sectional data. While we hypothesised that EFs may be a mechanism by which children are able to display behaviour differently across environments, it could be that adaptive functioning differences themselves underlie the observations that teachers make about children’s EF, for example, that informants are providing data about similar underlying behaviours when providing reports on both domains. Finally, it is important to note that our results may not generalise to all members of the autistic population, given that our sample was comprised only of early diagnosed autistic children and had a low prevalence of girls as well as minority children.

Given that clinical guidelines for the assessment of psychosocial functioning in children are to incorporate information from multiple informants, understanding the ways in which informants separately provide domain-relevant information, and what discrepant reporting can tell us, is important for guiding clinical practice. Our findings indicate that autistic children across levels of adaptive function as rated by their parents may present differently in the school environment, and this is associated with other child characteristics. Domains such as EF and IQ could be useful clinical tools in the identification and interpretation of children with discrepant reports across contexts. They could also be useful assessments for identifying environments in which autistic children may be likely to show strengths in their adaptive functioning abilities.

## Supplemental Material

sj-docx-1-aut-10.1177_13623613251407310 – Supplemental material for Profiles of parent–teacher discrepancy on autistic children’s adaptive functioning Supplemental material, sj-docx-1-aut-10.1177_13623613251407310 for Profiles of parent–teacher discrepancy on autistic children’s adaptive functioning by Rachel Lees Thorne, Nicky Wright, Andres De Los Reyes, Isabel M Smith, Anat Zaidman-Zait, Lonnie Zwaigenbaum, Tracy Vaillancourt, Peter Szatmari, Teresa A Bennett, Eric Duku, Annie E Richard, Connor Kerns and Rachael Bedford in Autism
