# Supplementary material for: Profiles of parent–teacher discrepancy on autistic children’s adaptive functioning
Source: Autism. 2026 Jan 7;30(3):695–706. doi: 10.1177/13623613251407310 (PMC12923639; doi:10.1177/13623613251407310)
Supplement: sj-docx-1-aut-10.1177_13623613251407310 – Supplemental material for Profiles of parent–teacher discrepancy on autistic children’s adaptive functioning [file sj-docx-1-aut-10.1177_13623613251407310.docx]

**Supplementary Materials**

**Profiles of parent-teacher discrepancy on autistic children’s adaptive functioning**

**Missing data**

We imputed missing data on socioeconomic status (SES), a covariate in our analyses using chained equations in STATA. The imputation model for SES included all analysis variables as well as an earlier measurement of household income (completed at child age 4), that contained more available data and was highly correlated (0.7) with the available data on household income at children’s current ages. Fifty imputed datasets were created, based on the fraction of missing information (von Hippel et al., 2018), these were pooled using Rubin’s rules (Little & Rubin, 2019). Only complete/observed data on predictors of interest (parent and teacher reports of EF) were used in analyses, due to a lack of auxiliary variables that were highly correlated with these variables, as is recommended for multiple imputation (Hardt et al., 2012). Most children in the lower adaptive functioning profile (96%), and some children in the intermediate adaptive functioning profile (31%) did not complete the WISC. This is likely due to the verbal nature of this assessment meaning that it was not an appropriate assessment of cognitive function in children with limited speech. Imputing this data would thus be inappropriate due to the strong likelihood of this data being missing not at random (MNAR; where data are systematically missing, such that the value of the missing variable is related to the reason that is it missing; Salgado et al., 2016).

S Table 1. Model fit statistics for latent class solution

|  | 2-class | 3-class | 4-class | 5-class |
| --- | --- | --- | --- | --- |
| a-BIC | 9077.95 | 8775.22 | 8733.03 | 8676.70 |
| LMR-LRT | .038 | <.001 | .35 | .309 |
| Entropy | .89 | .93 | .90 | .91 |

a-BIC = sample-size adjusted Bayesian Information Criterion, smaller values indicate better model fit; BLRT = Bootstrap Likelihood-Ratio Test, LMR = –Lo-Mendell-Rubin test. Both tests compare the k_0_ class model with the k_-1_ class model. Significant *p*-values indicate improvement with the addition of an extra class. Entropy – varies from 0-1; indicates how accurately the model defines classes. There is no universally accepted cut-off criterion, however above 0.8 is generally deemed to be acceptable

S Table 2. Model estimates for covariates in latent profile analysis model

|  | Lower AF- parent higher | Intermediate AF | Higher AF | Higher AF- teacher higher |
| --- | --- | --- | --- | --- |
| Halifax | -0.274, *p*= .794 | -0.330, *p*= .751 | 2.611, *p*= .020 | Reference profile |
| Hamilton | 0.696, *p*= 0.392 | 0.586, *p*= .449 | 1.662, *p*= .170 | - |
| Vancouver | -1.403, *p*= .097 | 0.485, *p*= .459 | 1.109, *p*= .268 | - |
| Edmonton | -0.100, *p*= .875 | 0.146, *p*= .814 | 0.509, *p*= .581 | - |
| Assessment timepoint | .994, *p*= .103 | 0.285, *p*= .564 | -0.298, *p*= .665 | - |
